# BAFF as a Key Modulator of Respiratory Mucosal B Cell Immunity in Viral Infection and Mucosal Vaccination

**DOI:** 10.3390/cells15131140

**Published:** 2026-06-23

**Authors:** Wael Alturaiki

**Affiliations:** Department of Medical Laboratory Sciences, College of Applied Medical Sciences, Majmaah University, Majmaah 11952, Saudi Arabia; w.alturaiki@mu.edu.sa

**Keywords:** BAFF, mucosal immunity, respiratory viral infection, IgA, airway B cell responses, iBALT

## Abstract

Mucosal immunity in the respiratory tract provides the first line of defense against airborne pathogens, yet most current vaccines fail to induce strong and durable immune responses at these sites. Respiratory viruses, including respiratory syncytial virus (RSV), influenza viruses, and coronaviruses, remain major global health threats, in part due to their ability to evade long-term mucosal protection. Although systemic vaccination generates robust circulating immunity, it induces limited local responses, particularly secretory immunoglobulin A (IgA), which is critical for preventing viral entry and transmission at the airway surface. The mechanisms regulating B cell responses within the airway mucosa are not fully understood. B cell–activating factor (BAFF), a member of the tumor necrosis factor (TNF) superfamily, has emerged as an important context-dependent regulator of mucosal B cell immunity. BAFF is produced by airway epithelial cells and multiple myeloid populations, including dendritic cells and neutrophils, and is rapidly induced during respiratory viral infection through type I interferon–dependent pathways. Functionally, BAFF supports B cell survival, differentiation, and class-switch recombination, promoting the generation of antibody-secreting plasma cells and enhancing IgA production. In the lung, these effects align with early, intermediate, and late stages of the response, supporting initial local antibody production, the formation of inducible bronchus-associated lymphoid tissue (iBALT), and the development of tissue-resident memory B cells that sustain long-term immunity. Although BAFF plays an essential role in mucosal immunity, its activity requires tight regulation to maintain immune balance. Current evidence supports BAFF as a promising immunomodulatory component and highlights its potential as an adjuvant platform for enhancing mucosal vaccine efficacy, warranting further investigation as a potential adjuvant in this context.

## 1. Introduction

Mucosal surfaces of the respiratory tract represent the principal portal of entry for clinically important respiratory viruses, including RSV, influenza viruses, and coronaviruses, which together impose a substantial global burden of morbidity and mortality, particularly among infants, older adults, and immunocompromised individuals [1,2]. At these barrier sites, the epithelial immune interface coordinates rapid innate sensing with activation of adaptive immune responses, forming an integrated defense system that limits viral replication, preserves tissue integrity, and reduces transmission [1,2]. Local humoral immunity at the airway mucosa plays a central role in this process, with tissue-resident B cells and locally produced antibodies providing rapid protection upon re-exposure [3,4,5]. However, natural infection rarely induces sterilizing or durable mucosal immunity, and reinfections with RSV and coronaviruses remain common throughout life [2,6,7].

A major limitation of current vaccination strategies is their inability to fully recapitulate this protective mucosal landscape. While parenteral vaccines elicit strong systemic immunity, they generate relatively weak and short-lived responses at the respiratory mucosa. For example, intramuscular mRNA vaccination against SARS-CoV-2 induces robust circulating neutralizing antibodies and memory lymphocytes but results in limited antibody and memory B cell responses within the bronchoalveolar compartment, where IgG predominates over secretory immunoglobulin A (IgA) [3,8,9]. In contrast, early induction of mucosal IgA correlates with improved viral control and reduced susceptibility to infection [4,5,7,8]. Secretory IgA, transported across epithelial barriers via the polymeric immunoglobulin receptor, mediates immune exclusion by preventing viral attachment and entry at the luminal surface [4,8,10]. These observations underscore a fundamental challenge in respiratory immunology, how to develop strategies that elicit durable, site-specific B cell responses capable of maintaining sustained IgA production at mucosal surfaces [4,8,9,10].

B cell-mediated immunity is fundamental to mucosal defense, supporting local antibody production, tissue-resident memory B cells, and rapid recall responses. The cytokine BAFF, a member of the TNF superfamily, is a key regulator of B cell survival and differentiation across both naïve and antigen-experienced compartments [11,12]. BAFF is produced by multiple myeloid and stromal populations, including dendritic cells and macrophages, and supports B cell proliferation and T cell–independent antibody responses [9,11,12]. In the respiratory tract, airway epithelial cells represent an additional infection-responsive source, as viral stimuli such as RSV upregulate epithelial BAFF production both in vivo and in vitro [6,13]. Type I interferons and pattern recognition receptor signaling, including Toll-like receptor (TLRs) and MAVS-dependent pathways, drive cell and tissue specific BAFF induction during viral infection [11,13]. BAFF plays a central role in B-cell homeostasis and immune responses, and its dysregulation has been associated with both impaired immunity and autoimmune disease [14].

Furthermore, beyond its role in host defense, BAFF is also strongly implicated in immune-mediated and lymphoproliferative disorders. Elevated BAFF levels are consistently reported in autoimmune diseases such as systemic lupus erythematosus, rheumatoid arthritis, and Sjögren’s syndrome, where excessive BAFF signaling supports the survival of autoreactive B cells and is associated with increased autoantibody production [15]. BAFF overexpression is likewise linked to B-cell malignancies, including multiple myeloma and certain lymphomas, in which BAFF acts as a survival and growth factor for tumor B cells, contributing to their persistence and proliferation [16]. Together, these findings highlight the dual nature of BAFF as both a key regulator of normal protective immunity and a potential driver of pathological B-cell responses in autoimmunity and cancer [15,16].

BAFF expression is consistently upregulated during acute respiratory viral infections and is associated with enhanced local B cell activation and antibody production, including early T-independent IgG and IgA responses [6,9,11]. The related cytokine APRIL cooperates with BAFF to promote class-switch recombination and support the survival of IgA-secreting plasma cells, thereby sustaining mucosal antibody responses [9,10,12]. These processes occur within specialized lymphoid structures such as inducible bronchus-associated lymphoid tissue, which provides a local niche for B cell activation, germinal center reactions, and memory formation [1,2,17].

Emerging evidence indicates that BAFF regulation in the lung is highly dynamic and context dependent, varying across stages of infection, age groups, and microanatomical niches, with roles in both protective immunity and immunopathology [6,11,12,13]. Understanding this balance is essential for harnessing BAFF-driven pathways in mucosal vaccine design while avoiding dysregulated B cell activation. This review examines the regulation and functional role of BAFF during respiratory viral infection, with a focus on its contribution to mucosal B cell responses and IgA production within the lung microenvironment, and explores its potential as a target for immunomodulation and vaccine adjuvant development. Within this context, BAFF is considered a context-dependent modulator linking protective mucosal immunity with immunopathology, with its effects shaped by its spatial, temporal, and quantitative regulation within the lung.

### Methods of Literature Search

A narrative review of the published literature was conducted using PubMed and Web of Science. Search terms included: “BAFF,” “B cell-activating factor,” “APRIL,” “mucosal immunity,” “respiratory virus,” “IgA,” “iBALT,” “airway B cell,” and combinations thereof. The search covered publications from 2000 to 2025. Non-English articles were excluded. Original research articles, review articles, and relevant preclinical and clinical studies were considered. Studies were selected based on their relevance to BAFF biology in the respiratory tract, mucosal B-cell responses, respiratory viral infections, and vaccine immunology.

## 2. Induction and Dynamic Regulation of BAFF in Airway Epithelial Cells and the Lung Microenvironment During Viral Respiratory Infection

Airway epithelial cells are increasingly recognized as active immune sentinels that couple viral sensing to the orchestration of local humoral immunity. Upon infection with respiratory viruses such as Respiratory syncytial virus, Influenza, and Rhinovirus infection, epithelial cells detect viral RNA primarily through endosomal Toll-like receptor 3 and cytosolic RIG-I–like receptors. This leads to activation of interferon regulatory factors and NF-κB, resulting in robust type I interferon production [18,19,20]. For example, respiratory syncytial virus infection upregulates Toll-like receptor 3 and enhances epithelial responsiveness to double-stranded RNA, amplifying downstream antiviral and inflammatory responses [18].

Within this antiviral program, epithelial cells also induce expression of BAFF, positioning the airway mucosa as a key interface linking innate viral recognition to B cell activation and antibody production [21,22]. The capacity of airway epithelium to produce BAFF was first demonstrated in human bronchial epithelial cells stimulated with interferon-β, where BAFF expression was strongly upregulated [21]. In our previous study, this observation was extended to in vivo viral infection, where BAFF levels were markedly increased in BAL samples and bronchial brushings during severe infant respiratory syncytial virus bronchiolitis [22]. Furthermore, we demonstrated that in primary pediatric airway epithelial cells and epithelial cell lines, BAFF expression peaks early after infection, consistent with its role as an interferon-responsive gene [22]. Importantly, blockade of interferon-β signaling or viral entry abrogates BAFF induction, whereas interferon-β alone is sufficient to drive BAFF expression, establishing a direct link between type I interferon signaling and epithelial BAFF production [21,22].

Similar interferon-driven mechanisms have been observed in other epithelial tissues, including salivary gland epithelial cells, where Toll-like receptor 3 activation and viral stimulation induce BAFF through both interferon-dependent and independent pathways [23,24]. These findings support a model in which viral RNA sensing activates interferon signaling and JAK/STAT pathways, leading to transcriptional induction of BAFF, with potential additional contributions from NF-κB–dependent signaling [13,21,23].

BAFF expression in the airway epithelium is temporally dynamic. In infected epithelial cells, BAFF expression rises rapidly and subsequently declines, reflecting an early interferon-driven response that may be followed by secondary regulation by inflammatory mediators [22]. Consistent with our findings, BAFF can be detected throughout the respiratory tract during acute viral infection, including upper airway secretions from children with respiratory syncytial virus, metapneumovirus, influenza H1N1, bocavirus, and rhinovirus, suggesting that repeated or sequential infections may sustain BAFF exposure at mucosal surfaces [22].

In early life, baseline epithelial BAFF is relatively low but is potently inducible by viral stimuli, emphasizing a developmentally regulated but rapidly activatable BAFF/BAFF-R axis in the pediatric airway [13]. Although airway epithelial cells are a dominant local source of BAFF during respiratory viral infection, other cell types contribute. Dendritic cells in the upper respiratory mucosa produce BAFF in response to TLR3 ligands and enhance T cell independent class-switch recombination to IgG and IgA in mucosal B cells [25]. Myeloid and stromal cells, including those in chronically inflamed tissues, also generate BAFF in response to IFNs and TLR stimulation, supporting a distributed network of BAFF production that reinforces B cell survival and activation [16,23,24].

Across lung compartments, BAFF shows distinct distribution, with levels detectable in both upper and lower airway secretions during acute viral infection [22,26]. Our in vivo RSV studies demonstrate broad BAFF expression throughout lung tissue, whereas CXCL13 is restricted to cell-rich regions associated with developing lymphoid aggregates, supporting coordinated BAFF–chemokine signaling in iBALT formation [26]. In chronic inflammatory lung disease, BAFF becomes concentrated within peribronchial and perivascular lymphoid aggregates. In COPD, BAFF antagonism prevents cigarette smoke–induced lymphoid follicle formation and immunoglobulin production in mice [27].

Mechanistically, multiple microenvironmental signals converge to regulate BAFF expression. IFN-β not only drives BAFF expression but also promotes its proteolytic release, reinforcing epithelial–B cell communication during infection [28]. In fibrotic settings, IL-1β and IL-17A act as upstream regulators of BAFF, with Gr1^+^ myeloid cells serving as a major source in inflamed lungs [27]. Across these contexts, BAFF interacts with lymphoid-organizing chemokines, including CXCL13 and CCL21, which are associated with well-developed iBALT [29,30].

## 3. BAFF and Regulation of Airway B Cell Responses

BAFF is a pivotal organizer of airway B cell immunity, integrating survival, activation, and differentiation signals with tissue-derived innate cues. Through its receptors, BAFF-R, TACI, and BCMA, it regulates both the size and functional quality of the B cell pool participating in mucosal responses. Among these, BAFF-R is the principal receptor sustaining transitional and follicular B cell survival through alternative NF-κB and PI3K/AKT–mTOR signaling pathways [31,32].

### 3.1. BAFF and APRIL: Shared Receptors, Overlapping and Distinct Functions

BAFF and its structural homologue APRIL (a proliferation-inducing ligand) share the receptors TACI and BCMA, and both promote IgA class-switch recombination and plasma cell survival at mucosal surfaces [33,34,35]. However, APRIL does not bind BAFF-R, which is uniquely activated by BAFF and is critical for naïve and transitional B cell survival [36,37]. Conversely, APRIL binds heparan sulfate proteoglycans independently of BAFF, allowing it to access distinct proteoglycan-rich niches [33,38,39]. At the functional level, both cytokines sustain IgA-secreting plasma cells through TACI and BCMA signaling, but BAFF additionally provides the survival threshold for entering mature follicular and marginal zone compartments [36,37,40]. Dual BAFF/APRIL blockade, exemplified by povetacicept, therefore produces a more complete B cell suppression than targeting either alone, as demonstrated in autoimmune disease models [37,41]. The comparative roles of BAFF and APRIL in mucosal B cell immunity are summarized in Table 1.

### 3.2. BAFF Receptors and Signalling

TACI and BCMA exhibit more restricted expression patterns, being upregulated on activated B cells, plasmablasts, and plasma cells [49]. High-order BAFF oligomers preferentially signal through TACI and BCMA, potently supporting plasmablast and plasma cell survival [50]. Memory B cells also express BAFF-R and rely on BAFF signaling through IKK2 for long-term survival and optimal recall responses [51]. Within the airway microenvironment, epithelial and myeloid cells serve as key inducible sources of BAFF [48]. In allergic airway inflammation and virus-induced asthma exacerbations, macrophages and other myeloid cells in BAL upregulate BAFF, APRIL, and their receptors [37]. Pharmacologic inhibition of BAFF in this context reduces CD19^+^CD27^+^ and IgE^+^ B cell subsets and improves lung function in mouse models [48].

Beyond its role in B cell survival, BAFF is a critical driver of immunoglobulin class-switch recombination (CSR) and antibody secretion, particularly IgA at mucosal surfaces [45]. BAFF and APRIL engagement of BAFF-R and TACI enables CD40-independent, T cell–independent CSR, allowing early diversification of the antibody response before the establishment of full T cell help [52]. Mechanistically, TACI signals through a pathway involving MyD88, IRAK, and TRAF6, directly linking innate immune signals to AID expression and germline CH transcription in mucosal B cells [52].

These BAFF-dependent processes are tightly integrated with the generation and maintenance of airway IgA-secreting plasma cells and tissue-resident memory B cells, which underpin durable mucosal immunity [53]. BAFF and APRIL signalling through TACI and BCMA are essential for the long-term maintenance of memory plasma cells within specialized survival niches, preventing endoplasmic reticulum stress–induced apoptosis and sustaining continuous antibody secretion [37]. The diverse roles of BAFF in airway homeostasis, antiviral defense, mucosal immune organization, and chronic lung disease are summarized in Table 2.

**Figure 1:**
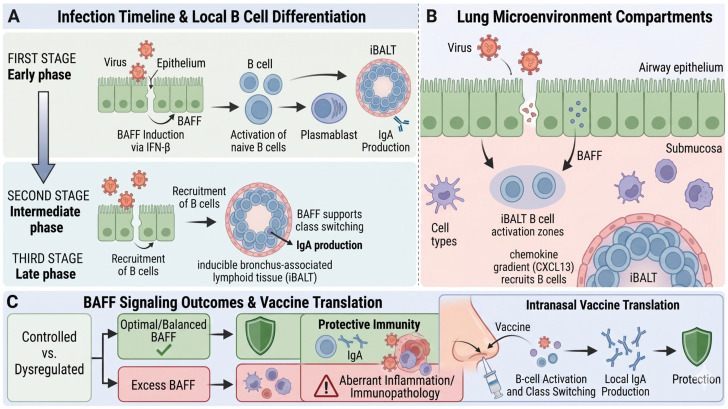
BAFF-mediated regulation of pulmonary B-cell responses during respiratory viral infection and its implications for mucosal vaccine translation. (**A**) During respiratory viral infection, epithelial-derived BAFF promotes B-cell activation, plasmablast differentiation, local IgA production, and the formation of inducible bronchus-associated lymphoid tissue (iBALT). (**B**) Within the pulmonary microenvironment, BAFF and CXCL13 support B-cell recruitment, activation, survival, and organization within iBALT, facilitating local humoral immune responses. (**C**) Optimal BAFF signaling promotes B-cell activation, class switching, and local IgA production, resulting in protective mucosal immunity, whereas excessive BAFF may contribute to immunopathology. During intranasal vaccination, BAFF-mediated B-cell responses may enhance local IgA production and protection against respiratory pathogens, highlighting the potential of BAFF-targeted strategies for mucosal vaccine development. The image was generated using ImageGen v2.0.

Collectively, these findings establish BAFF as a key modulator of airway B cell immunity. By coordinating survival, class switching, plasma cell differentiation, and memory formation, BAFF integrates epithelial, complement, and interferon-driven signals to shape rapid, IgA-dominated humoral responses at the respiratory mucosa. The mechanistic framework underpinning these processes, including the temporal stages of infection, lung microenvironment compartments, and vaccine translation implications, is illustrated in Figure 1.

## 4. BAFF in Immunopathology of the Lung

BAFF is a pivotal survival and differentiation factor for B cells, and dysregulated BAFF expression in the lung drives pathogenic humoral and inflammatory responses that contribute to chronic airway diseases. Excess BAFF relaxes peripheral tolerance thresholds, rescuing autoreactive and low-affinity B cells from apoptosis and promoting autoantibody production through BAFF receptor–dependent activation of canonical and noncanonical NF-κB pathways [16,68]. Excessive BAFF expression has been shown to drive autoimmune manifestations consistent with SLE and Sjögren’s syndrome, underscoring the importance of tightly regulated BAFF signaling [14].

In human disease, these mechanisms are reflected in consistently elevated BAFF levels observed in SLE, rheumatoid arthritis, and Sjögren’s syndrome, where sustained BAFF signaling promotes the survival of autoreactive B cells and drives pathogenic autoantibody production [44,69]. Increased BAFF expression has also been documented in B cell malignancies including multiple myeloma and certain lymphomas [70]. The clinical significance of this pathway is underscored by belimumab, an anti-BAFF monoclonal antibody approved for SLE and lupus nephritis, and atacicept (dual BAFF/APRIL inhibitor), which is under investigation for SLE and IgA nephropathy [69,70,71].

Within the lung, BAFF-driven survival of naïve and germinal center-like B cells promotes ectopic lymphoid neogenesis. In severe COPD, BAFF is markedly upregulated within pulmonary lymphoid follicles; BAFF expression correlates with follicle size, reduced B cell apoptosis, and airflow limitation, supporting a self-perpetuating loop of B cell survival and follicular expansion [61]. BAFF antagonism using BAFF-R:Fc during chronic cigarette smoke exposure reduces pulmonary B cell numbers, prevents lymphoid follicle formation, lowers local immunoglobulin levels, and attenuates emphysematous destruction [27].

Similar BAFF-dependent B cell hyperplasia contributes to interstitial lung disease in common variable immunodeficiency (CVID), where elevated BAFF derived from monocytes promotes BAFF receptor-dependent Bcl-2 upregulation and accumulation of naïve B cells within tertiary lymphoid structures. Although B cell depletion transiently improves disease, recurrence parallels rising BAFF levels, implicating BAFF as a driver of chronic pulmonary autoimmunity [66]. In fibrotic lung disease, BAFF is induced downstream of IL-1β and IL-17A in bleomycin models, and BAFF blockade limits TGF-β production, collagen deposition, and IL-17A–dependent fibrosis [65].

Furthermore, the immunological effects of BAFF are highly context-dependent and are influenced by three key factors: the duration of expression, the cellular source, and the surrounding inflammatory environment. Transient BAFF induction during acute infection supports B-cell survival and protective antibody responses, including mucosal IgA production, whereas sustained BAFF expression in chronic inflammatory conditions promotes autoreactive B-cell survival, ectopic lymphoid follicle formation, and persistent tissue inflammation [61,72]. Epithelial-derived BAFF induced through interferon signaling during infection is generally self-limiting and linked to antiviral host defense, whereas chronic production by monocytes, macrophages, and stromal cells may perpetuate immune dysregulation [28,73]. The inflammatory milieu further shapes BAFF activity; in the presence of cytokines such as IL-17A and TGF-β, BAFF can promote tertiary lymphoid structure formation and amplify pathological immune responses [72]. Collectively, these factors determine whether BAFF contributes to protective immunity or chronic inflammation.

Moreover, BAFF dysregulation is also observed in immunocompromised populations. In HIV infection, chronic immune activation is associated with elevated circulating BAFF levels that correlate with B-cell hyperactivation, hypergammaglobulinemia, and impaired memory B-cell function [66,74]. Similarly, patients with common variable immunodeficiency (CVID) exhibit altered BAFF signaling, and excessive BAFF activity has been linked to B-cell dysregulation and progressive pulmonary lymphoid disease [66,75]. These findings highlight the importance of host immune status when considering BAFF-targeted therapies or BAFF-enhancing vaccine strategies, as excessive BAFF signaling may exacerbate immune dysfunction rather than improve protective immunity [66,76].

BAFF also promotes allergic airway pathology. In an OVA-RSV asthma model, BAFF derived from pulmonary macrophages sustains BAFF receptor and TACI expressing B cells and enhances IgE production, whereas pharmacologic inhibition of BAFF signaling reduces IgE levels, inflammatory cytokines, and airway inflammation [63].

Collectively, these findings position BAFF as a key driver of pathogenic B cell survival, ectopic lymphoid structure formation, cytokine amplification, and fibrosis in the lung, providing a mechanistic rationale for targeting BAFF or BAFF/APRIL pathways as potential therapeutic strategies in BAFF-high chronic pulmonary diseases, while emphasizing the need for precise modulation to preserve protective mucosal immunity.

## 5. Implications of BAFF for Mucosal Vaccine Development

Systemic intramuscular vaccines effectively prevent severe disease but are less efficient at inducing durable immunity at mucosal entry sites, where protection depends on local IgA, tissue-resident B cells, and organized mucosa-associated lymphoid structures. In contrast, mucosal vaccines delivered through the respiratory tract elicit stronger local IgA responses and tissue-resident immunity, highlighting the need for adjuvants that specifically enhance mucosal humoral responses [77,78,79,80,81]. Within this framework, BAFF emerges as a compelling immunomodulator due to its roles in B-cell survival, class-switch recombination, antibody production, and T-cell-independent mucosal IgA responses [82].

BAFF signals through BAFF-R, TACI, and BCMA to activate canonical and alternative NF-κB pathways that sustain B-cell survival and promote IgA class-switch recombination [58]. Elevated BAFF levels in Peyer’s patches have been associated with expansion of IgA germinal-center B cells [58]. Neutrophil-derived BAFF downstream of complement receptor signaling is essential for germinal-center IgA class switching in Peyer’s patches and for fecal IgA production [58].

Evidence from vaccination models suggests that BAFF can function as a molecular adjuvant. In an HIV-1 Env study, gp140 fused to BAFF or APRIL modestly enhanced B cell proliferation and antigen-specific IgG in mucosal secretions compared with gp140 alone [83]. However, direct evidence supporting its efficacy in mucosal vaccine settings remains limited. Specifically, no study to date has demonstrated that exogenous BAFF administration enhances protective mucosal IgA responses against a respiratory virus in vivo. The available data come from four distinct contexts, none of which directly addresses this question: (i) an HIV-1 gp140 model in which BAFF fusion modestly increased mucosal IgG but not IgA [83]; (ii) a *Pseudomonas aeruginosa* model in which BAFF overexpression improved systemic protection, with no assessment of mucosal IgA [84]; (iii) an in silico multi-epitope construct with no in vivo validation [85]; and (iv) rabies virus particles incorporating membrane-anchored BAFF, tested in a non-respiratory, non-mucosal setting [86]. Taken together, these studies demonstrate that BAFF can amplify antigen-specific antibody responses in various vaccine formats, but none establishes that it can induce protective mucosal IgA in the respiratory tract (Table 3). Dedicated preclinical studies evaluating safety, dosing, and intranasal delivery routes are therefore required before any translational application can be considered.

Experimental studies have explored BAFF as a vaccine adjuvant demonstrating its ability to enhance antigen-specific antibody responses. Specifically, it has been reported that BAFF overexpression enhanced protective immunity in a *Pseudomonas aeruginosa* vaccine model [86], while other study demonstrated that membrane-anchored BAFF incorporated into rabies virus particles accelerated neutralizing antibody responses [86]. These provide direct experimental evidence, though the mechanisms and safety considerations remain incompletely understood.

Additionally, evidence supports a role for BAFF in promoting mucosal antibody responses, particularly in settings with limited or delayed T cell help [11,58]. BAFF-driven IgA responses have important implications for airway protection: intranasal vaccines that induce high mucosal IgA titers provide superior control of respiratory viruses [77,78,79,80,81].

Moreover, BAFF may contribute to the formation and maintenance of local lymphoid structures. Intratracheal vaccination can induce iBALT, supporting germinal center activity and long-lived plasma cells in the lung [79]. In addition, mucosal adjuvants that induce IL-1β in airway epithelium promote chemokine expression, immune cell recruitment, and BAFF upregulation, linking epithelial activation to tissue-resident lymphocyte networks and durable immunity [87].

However, translational use of BAFF must balance immune enhancement with safety. Chronic BAFF/APRIL overexpression promotes autoreactive B cell survival and autoantibody production, contributing to diseases such as SLE, while dual BAFF/APRIL blockade suppresses B cell proliferation and immunoglobulin levels [41,88]. BAFF functions as a context-dependent modulator of B cell homeostasis: excessive signaling drives pathology, whereas inhibition contracts humoral immunity. Practical implementation also faces challenges related to large-scale production, cost, formulation stability, and potential variability in reactogenicity. Effective vaccine strategies will therefore require transient, localized BAFF induction to enhance mucosal responses without disrupting systemic tolerance.

**Table 3:** Evidence for BAFF as a Vaccine Adjuvant: Summary of Key Studies.

Study	Model/Antigen	Route/Format	Key Outcome	Limitation/Gap
Tertilt et al., 2009 [84]	*Pseudomonas aeruginosa* vaccine; BAFF overexpression in mice	Systemic (parenteral)	BAFF overexpression enhanced vaccine-induced antibody responses and significantly improved survival after bacterial challenge	Not a mucosal vaccine; no IgA endpoint assessed; non-respiratory pathogen
Kanagavelu et al., 2014 [89]	HIV-1 Gag; adenoviral vector (Ad5) co-expressing multi-trimeric SP-D-BAFF (mice)	Systemic (Ad5 vector)	SP-D-BAFF significantly reduced vaccinia-Gag replication in female genital tract; enhanced T cell-mediated anti-viral immunity	Primarily T cell outcome; no mucosal IgA measured; non-respiratory model
Gupta et al., 2015 [90]	HIV-1 gp140 DNA vaccine; SP-D-BAFF and SP-D-APRIL as molecular adjuvants (mice)	Systemic (DNA + protein boost)	SP-D-BAFF significantly enhanced anti-gp120 IgG, germinal center responses, and HIV-1 tier 1 and tier 2 neutralizing antibody titers; enhanced antibody avidity	Systemic model only; no mucosal IgA endpoint; not a respiratory vaccine
Chen et al., 2019 (Antiviral Res) [91]	Influenza H5N1 VLPs molecularly fused with BAFF or APRIL; also multi-subtype H5H7 and H1H5H7 BAFF-VLPs (mice)	Parenteral (VLP + alum)	BAFF-VLPs with alum elicited broadly neutralizing IgG against homologous and two heterologous H5N1 clades; conferred protective immunity against live H5N1 challenge; multi-subtype VLPs induced cross-reactive neutralizing antibodies	Parenteral route only; no intranasal or mucosal IgA data; alum dependency limits standalone BAFF effect interpretation
Liu et al., 2020 [83]	HIV-1 trimeric gp140 fused with BAFF, APRIL, or CD40L (mice)	Parenteral (protein fusion)	Modestly enhanced antigen-specific IgG in mucosal secretions vs. gp140 alone; increased B cell proliferation	No IgA endpoint; not a respiratory or mucosal vaccine; modest effect size
Plummer & McGettigan, 2019 [86]	Rabies virus particles incorporating membrane-anchored BAFF (mice)	Parenteral (VLP)	Accelerated and improved neutralizing antibody responses; enhanced B cell activation vs. standard RABV vaccine	Non-respiratory, non-mucosal setting; IgA not assessed
Alnajran et al., 2025 [85]	RSV multi-epitope construct incorporating BAFF and APRIL (in silico reverse vaccinology)	Computational/in silico	Predicted enhanced immunogenicity, antigenicity, and immune activation profiles; integration of BAFF/APRIL improved in silico vaccine scores	No in vivo validation; computational predictions require experimental confirmation
Xie et al., 2025 (Front Immunol) [92]	BCG vaccination followed by post-vaccination BAFF or APRIL immunotherapy (mice; Mtb pulmonary challenge)	Subcutaneous BCG + i.p. BAFF/APRIL (transient)	BCG + BAFF/APRIL immunotherapy enhanced marginal-zone B cells, plasma cell differentiation, and central memory T cells; superior long-term pulmonary protection vs. BCG alone	Post-vaccination immunotherapy rather than adjuvant co-formulation; pulmonary (not airway mucosal) protection endpoint; non-viral pathogen

## 6. Future Directions

Future research should focus on defining how BAFF can be harnessed to enhance protective mucosal immunity while avoiding excessive or prolonged activation that may promote immunopathology. Strategies aimed at localizing BAFF activity within the respiratory tract, including targeted delivery systems, BAFF-based immunomodulators, and antigen-directed approaches, may provide opportunities to strengthen IgA responses and tissue-resident immune memory while minimizing systemic effects.

Advances in vaccine design are expected to facilitate the incorporation of BAFF and related signaling pathways into next-generation mucosal vaccine platforms. In addition, combination approaches that integrate BAFF-inducing signals with other innate immune pathways may improve coordination between epithelial, myeloid, and lymphoid compartments, thereby enhancing local immune responses. Future translational studies should prioritize human-relevant models and carefully evaluate the magnitude, duration, and localization of BAFF signaling to identify safe and effective strategies for therapeutic and vaccine development.

## 7. Conclusions

BAFF has emerged as a central regulator of respiratory mucosal immunity, linking innate immune sensing to adaptive B-cell responses. Its rapid induction following viral infection promotes local B-cell activation, IgA production, and the generation of tissue-resident memory populations that contribute to protection at mucosal surfaces. Furthermore, spatial and temporal variations in BAFF expression influence the formation of iBALT, germinal center activity, and local antibody production within the lung. Despite its important protective functions, BAFF activity must be tightly regulated. Excessive or prolonged signaling can disrupt B-cell tolerance, promote ectopic lymphoid tissue formation, and contribute to chronic inflammatory and autoimmune diseases of the respiratory tract. These observations highlight the dual nature of BAFF as both a mediator of protective immunity and a potential driver of immunopathology.

Collectively, current evidence supports a model in which BAFF functions as an immunological rheostat whose biological effects depend on the magnitude, duration, and anatomical localization of its activity. A deeper understanding of the cellular and molecular mechanisms that regulate BAFF within the respiratory tract will be essential for the development of future therapeutic and vaccine strategies. By enabling precise modulation of mucosal B-cell responses, BAFF represents a promising target for improving protection against respiratory pathogens while maintaining immune homeostasis.

## Figures and Tables

**Table 1:** Comparative Roles of BAFF and APRIL in Mucosal B Cell Immunity.

Feature	BAFF	APRIL	References
Primary receptors	BAFF-R, TACI, BCMA	TACI, BCMA	[34,42,43]
Main role in B-cell biology	B-cell maturation, survival, homeostasis	Plasma-cell survival, IgA-linked humoral responses	[35,37,44]
Effect on IgA responses	Supports IgA class switching (esp. via TACI/BAFF-R)	Potent inducer of TI IgA class switching	[34,45,46,47]
Sources in respiratory mucosa	Airway epithelial cells, DCs, macrophages, neutrophils	Same	[43,48]
Contribution to mucosal immunity	Maintains local B-cell populations and Ab responses	Sustains mucosal plasma cells and secretory IgA	[35,37,42]
Relevance during respiratory infection	Upregulated by viral/bacterial stimuli; enhances humoral immunity	Supports durable mucosal Ab and plasma cells	[14,22,28,43]

**Table 2:** BAFF in Regulation and Dysregulation of Airway B Cell Immunity and the Lung Microenvironment.

Setting	BAFF Source	BAFF Regulatory Role	Functional Outcome	Disease/Consequence	Key References
Acute RSV infection (infants/children)	Airway epithelial cells (IFN-β dependent); DCs; infiltration inflammatory immune cells	Rapidly induced via TLR3/JAK-STAT; supports early B cell activation and IgA CSR	Early IgA and IgG production; plasmablast formation; iBALT initiation	Protective mucosal humoral immunity; limits viral spread	[22,26,28,54]
Acute influenza and rhinovirus infection	Airway epithelial cells; upper airway DCs	TLR3-driven BAFF induction activates T-independent IgG and IgA CSR in mucosal B cells	IgA and IgG class switching; B cell activation; local antibody production	Protective mucosal immunity against multiple respiratory viruses	[11,25]
SARS-CoV-2/COVID-19 infection	Myeloid cells; epithelial cells	BAFF elevated during acute infection; correlates positively with total B cells and IgG+ plasmablasts	B cell activation; IgG plasmablast expansion; supports systemic humoral response	Protective antibody response; dysregulated in MIS-C (elevated BAFF with reduced BAFFR expression and autoantibody production)	[55,56]
Early life/pediatric airway (baseline and viral infection)	Airway epithelial cells (low baseline; virally inducible via JAK/STAT)	Developmentally low but rapidly activated BAFF/BAFF-R axis upon viral stimulation	Rapid induction of BAFF supports local B cell responses in pediatric airways	Protective; developmentally regulated defense mechanism	[13]
iBALT formation during respiratory infection	Epithelial cells; myeloid cells; stromal cells	BAFF together with CXCL13 organizes B cell niches; sustains GC activity and plasma cell survival	Germinal center formation; class-switched IgA and IgG; tissue-resident memory B cells	Long-term protective mucosal immunity; reduced systemic vaccine dependence	[26,30,57]
Neutrophil-complement axis (gut and lung IgA)	Neutrophils (C3aR1/C5aR1-dependent BAFF release)	Complement receptor signaling drives neutrophil BAFF production; enables T-independent IgA CSR	IgA germinal center B cell expansion in Peyer’s patches; fecal IgA; likely conserved in lung	Protective T-independent mucosal IgA; innate-adaptive bridge	[58]
Cystic fibrosis airways (*Pseudomonas aeruginosa*)	Airway epithelial cells; myeloid cells	BAFF elevated in CF airways irrespective of Pseudomonal infection; BAFF depletion worsens infection outcomes	B cell recruitment and differentiation; local antibody production against *P. aeruginosa*	Protective role suggested (depletion exacerbates infection); but chronic BAFF elevation may reflect ineffective clearance	[59,60]
COPD (cigarette smoke-induced)	Follicular stromal cells; B cells; macrophages	Markedly overexpressed in pulmonary lymphoid follicles; prevents B cell apoptosis via Bcl-2; drives ectopic follicle expansion	Lymphoid follicle formation; local immunoglobulin elevation; reduced B cell apoptosis; airflow limitation	Pathogenic: emphysema progression; BAFF antagonism (BAFF-R:Fc) reduces follicles, immunoglobulins, and lung destruction	[27,61]
BAFF and T lymphocytes in COPD	Lung structural and myeloid cells	BAFF directly promotes CD4+ and CD8+ T cell activation and survival; amplifies airway inflammation	Enhanced T cell-mediated airway inflammation; cytokine amplification loop	Pathogenic: BAFF-driven T cell hyperactivation contributes to COPD progression	[62]
Allergic airway inflammation/OVA-RSV asthma	Pulmonary macrophages	Sustains BAFF-R+ and TACI+ B cells; promotes IgE CSR; pharmacologic BAFF inhibition reduces IgE	IgE+ B cell expansion; elevated IgE; airway eosinophilia; increased B cell precursors in lung	Pathogenic: enhanced allergic response; IgE-mediated mast cell activation; BAFF-R blockade reduces eosinophils	[63,64]
Pulmonary fibrosis (bleomycin model/IPF)	Gr1+ myeloid cells; neutrophils (IL-1beta/IL-17A-driven)	BAFF induced downstream of IL-1beta and IL-17A; promotes TGF-beta, collagen deposition; elevated in IPF BAL	Fibrosis progression; collagen deposition; IL-17A-dependent pathology	Pathogenic: BAFF blockade limits TGF-beta and fibrosis; elevated BAFF in human IPF	[65]
CVID-associated interstitial lung disease	IFN-gamma-activated CD14+ monocytes (STAT1-dependent)	Monocyte-derived BAFF drives BAFF-R-dependent Bcl-2 upregulation; naïve B cell accumulation in tertiary lymphoid structures	B cell hyperplasia in TLS; elevated IgM; disease progression; transient improvement after B cell depletion before relapse	Pathogenic: BAFF-driven chronic pulmonary autoimmunity; recurrence parallels BAFF re-elevation	[66]
Adenovirus pneumonia (autoimmunity)	Pulmonary myeloid cells	High BAFF from myeloid cells promotes tertiary lymphoid structure formation in lower airways	Local autoantibody production; CD19+CD21low autoreactive B cell accumulation	Pathogenic: BAFF links severe viral pneumonia to autoreactive B cell responses	[67]
Rheumatoid arthritis-associated lung disease (RA-ILD)/iBALT	Stromal and myeloid cells within iBALT	BAFF together with CXCL13, CCL21, lymphotoxin sustains GC-like B cell follicles in iBALT	Tertiary lymphoid structure maturation; sustained autoantibody production	Pathogenic: iBALT with GC activity linked to RA-ILD progression	[29]

## Data Availability

AI-assisted tools were used for figure generation and language editing. All scientific concepts, interpretations, and conclusions are the original work of the author. No new data were created or analyzed in this study.
